# Impact of opioid use disorder on oxytocin required for labor induction in term pregnancies

**DOI:** 10.1002/pmf2.70490

**Published:** 2026-10-21

**Authors:** Hansika Kunduru, Judy Jiang, Hannah Hill, Jessica Wilder

**Affiliations:** ^1^ Department of Nutrition Case Western Reserve University Cleveland Ohio USA; ^2^ Department of Obstetrics and Gynecology Metro Health Medical Center Case Western Reserve University School of Medicine Cleveland Ohio USA

**Keywords:** amniotomy, labor induction, labor progression, opioid use disorder, oxytocin

## Abstract

**Objective:**

Oxytocin is a neuropeptide proven to impact human labor, lactation, and social affinity and is of interest in the study of pain and μ‐opioid receptor modulation. Individuals impacted by opioid use disorder (OUD) frequently have μ‐opioid receptor dysregulation. In this study, we sought to investigate the effect of OUD on the effectiveness of synthetic oxytocin in term inductions of labor.

**Study design:**

Matched retrospective cohort study of individuals with term pregnancies undergoing induction of labor with oxytocin from 2020 to 2024 at an urban safety‐net hospital. The total amount of oxytocin required to achieve vaginal delivery and total induction of labor time were compared between those with OUD and those without. The groups were matched by age, parity, gestational age, month of delivery, and body mass index. Wilcoxon signed‐rank tests were used to compare continuous variables between two groups.

**Results:**

This study evaluated 212 (106 without OUD and 106 with OUD) individual vaginal deliveries. There was no significant difference in total oxytocin dose used for induction between patients with OUD and those without OUD. While total induction time was longer in patients with OUD (median 710 vs. 590 min, *p* = 0.018), this difference was no longer significant after adjusting for Foley balloon use and age (*p* = 0.105). However, patients with OUD spent an estimated 66 min longer in induction after amniotomy than those without OUD (median 269 vs. 335 min, *p* = 0.015).

**Conclusion:**

Individuals with OUD were not found to have significantly different total required oxytocin doses to deliver than those without OUD. However, patients with OUD experienced significantly longer induction time following amniotomy compared to those without OUD. Further studies are needed to examine the impact of OUD on induction of labor with oxytocin.

## 1 INTRODUCTION

Induction of labor is one of the most common obstetric procedures in the United States and synthetic oxytocin (Pitocin) is a commonly used pharmacologic agent for labor induction because of its ability to stimulate uterine contractions and promote cervical dilation [1, 2]. However, the effectiveness of oxytocin in labor induction depends on the physiology of the mother and the response of her myometrial oxytocin receptor [2].

Opioid use disorder (OUD) has been rising among pregnant individuals. Studies have demonstrated a 400% increase in OUD documented at delivery hospitalizations over the past two decades [3]. Most of the literature regarding OUD in pregnancy has focused on neonatal outcomes and maternal complications associated with chronic opioid exposure [4]. However, the broad effects of OUD on physiological progression of labor remain poorly understood.

Oxytocin is a neuropeptide that is integral in uterine contractions and labor progression. It has two main roles: as a hormone that regulates myometrial contractions peripherally and as a central neuromodulator that controls reward and stress pathways [5]. Importantly, oxytocin also functions as a modulator of the endogenous opioid system. Studies have suggested that oxytocin mechanistically acts as a positive allosteric modulator on the μ‐opioid receptor, increasing signal transduction and downstream physiologic response [6]. Notably, chronic exposure to exogenous opioids may affect μ‐opioid receptor function and neuroendocrine regulation [7, 8]. Previous studies have suggested that chronic exposure to opioids contributes to lower levels of endogenous oxytocin, manifesting in a relative hypo‐oxytocinergic state [8]. These physiologic changes may influence labor progression, especially after amniotomy when endogenous oxytocin release normally accelerates uterine contractions and delivery [1].

Although these interactions have been documented, limited clinical data have evaluated the influence that OUD has on oxytocin's response to induce labor and its progression. Therefore, the purpose of this study was to determine whether OUD affects the total dose of synthetic oxytocin required for successful induction of labor in term pregnancies and to evaluate differences in labor progression between patients with and without OUD.

## 2 METHODS

We conducted a matched retrospective cohort study at an urban safety‐net hospital. Data were collected from January 1, 2020 to December 31, 2024 for pregnant patients with term deliveries who had their labor induced with synthetic oxytocin (Pitocin) and delivered vaginally. OUD status was identified via International Classification of Diseases, 10th Revision (ICD‐10) diagnosis codes in the electronic medical record. Patients enrolled in a medication‐assisted treatment program were assumed to have non‐active opioid use, as institutional protocol does not require urine drug screening on admission for patients participating in a structured medications for opioid use disorder (MOUD) program. Patients without a history of OUD served as controls and were matched 1:1 to cases using a nearest neighbor matching algorithm on maternal age, parity (nulliparous vs. multiparous), gestational age at delivery, month of delivery, and body mass index (BMI) to define the groups. Collected variables included maternal age, race, BMI, parity, gestational age, and delivery date.

The primary outcome was the total cumulative oxytocin dose used to achieve vaginal delivery. Oxytocin was administered as a 30 IU/500 mL solution; doses are reported in mL. In addition, we measured the progression of labor by calculating the time from the start of the induction process to delivery, time from admission to amniotomy, and time from amniotomy to delivery. We also recorded the use of cervical ripening methods including Foley balloon catheters and/or misoprostol. Statistical analyses were performed using R (R Foundation for Statistical Computing) in RStudio (Posit Software). Continuous variables are reported as medians with interquartile ranges (IQRs). The χ^2^ and Fisher's exact test were used to compare frequencies of categorical variables, and the Wilcoxon signed‐rank test was used to evaluate continuous variables between those with and without OUD. Statistical significance was defined as *p* < 0.05.

## 3 RESULTS

A total of 212 vaginal deliveries were included in the study. The study included 106 patients without OUD and 106 patients with OUD. The study population was predominantly White (85.8% control vs 87.7% OUD), with no significant demographic differences between the matched cohorts regarding racial distribution (*p* > 0.9; Table 1).

**TABLE 1:** Baseline demographic characteristics.

Characteristic	Non‐OUD (*n* = 106)	OUD (*n* = 106)	*p* value^a^
Maternal age (years)			0.5
Mean (SD)	30.2 (4.5)	30.7 (4.4)	
Median (Q1, Q3)	30.0 (26.9, 32.7)	30.2 (27.8, 33.7)	
Gestational age (weeks)			>0.9
Mean (SD)	38.92 (1.00)	38.98 (1.01)	
Median (Q1, Q3)	39.14 (38.29, 39.29)	39.14 (38.29, 39.57)	
Race, *n* (%)			>0.9
White	91 (85.8%)	93 (87.7%)	
Hispanic	11 (10.4%)	9 (8.5%)	
Black	4 (3.8%)	4 (3.8%)	
Asian	0 (0%)	0 (0%)	
Parity, *n* (%)			0.6
Nulliparous	13 (12%)	16 (15%)	
Multiparous	93 (88%)	90 (85%)	
BMI (kg/m^2^)			>0.9
Mean (SD)	31 (7)	31 (7)	
Median (Q1, Q3)	30 (26, 36)	30 (26, 35)	
Foley balloon, *n* (%)	22 (21%)	36 (34%)	0.030
Misoprostol, *n* (%)	8 (7.6%)	4 (3.8%)	0.2

Abbreviations: BMI, body mass index; OUD, opioid use disorder.

^a^ Fisher's exact test; Pearson's Chi‐squared test.

There was no significant difference found between the amount of oxytocin used to induce delivery between patients with OUD and patients without OUD. Patients without OUD had a median total oxytocin dose of 87 mL versus 103 mL in patients with OUD (*p* = 0.80; Table 2). In addition, there was no statistically significant difference in the time from admission to amniotomy between the OUD group and the control group (287 vs. 345 min, *p* = 0.4; Table 2).

**TABLE 2:** Non‐OUD versus OUD on oxytocin dose and induction time.

	Non‐OUD (*n* = 106)	OUD (*n* = 106)	*p* value^a^
Total induction time (minutes)	590 (446, 876)	710 (508, 1027)	0.018^b^
Oxytocin dose (in mL of 30 IU/500 mL solution)	87 (44,155)	103 (41,168)	0.8
Admission to amniotomy (minutes)	287 (208,473)	345 (211,523)	0.4
Amniotomy to delivery (minutes)	269 (172,425)	335 (229, 514)	0.015

Abbreviation: OUD, opioid use disorder.

^a^ Wilcoxon signed‐rank test.

^b^ Adjusted for Foley balloon use and age: *p* = 0.105.

The median total induction time was 590 min (IQR: 446–876) in patients without OUD versus 710 min (IQR: 508–1027) in patients with OUD (*p* = 0.018; Table 2). After adjusting for Foley balloon use and age, the difference in total induction time was no longer statistically significant (*p* = 0.105). However, there was a significant increase in the time from amniotomy to delivery in patients with OUD. Patients with OUD spent an estimated 66 min longer in labor following amniotomy. Patients with OUD had a median time from amniotomy to delivery of 335 versus 269 min in patients without OUD (*p* = 0.015; Table 2).

## 4 DISCUSSION

While previous studies have primarily focused on neonatal outcomes and maternal complications, there are limited data evaluating the physiologic progression of labor during induction in patients with OUD. The overall amount of oxytocin required to induce labor did not differ between groups; however, the time from amniotomy to delivery was significantly longer in patients with OUD. Although total induction time was also greater in the OUD group, this difference was no longer statistically significant after controlling for age and Foley balloon use.

The increased duration suggests the possibility of a physiologic difference in the progression of labor in patients with OUD. While the early stages of induction appeared similar between groups, patients with OUD did not demonstrate the accelerated progression of labor typically observed following amniotomy [1]. These differences may reflect alterations in μ‐opioid receptor signaling associated with chronic opioid exposure [7]. Because μ‐opioid receptors interact with the oxytocin system through allosteric modulation, opioid‐induced changes to μ‐opioid receptor pathways may negatively influence myometrial contractility during the phase of labor in which oxytocin‐mediated contractions are most important [6, 8]. Our findings therefore suggest that OUD may influence labor progression after amniotomy even though the overall oxytocin requirement remains unchanged.

Our predominantly White study population limits generalizability. Racial disparities in labor outcomes, including increased rates of cesarean delivery, have been reported in the literature, and by including only patients with vaginal deliveries, our sample may be skewed. Additionally, polysubstance use is common among patients with OUD, and whether other illicit substances that affect the μ‐opioid receptor contributed to the outcomes observed in this study remains unclear.

Future studies should include patients who failed induction as a comparison group and should stratify by race and BMI to clarify whether associations differ across subgroups.

A strength of this study includes the use of matched controls to account for key maternal and obstetric variables that may influence labor progression. Despite this strength, this research has limitations. Because this is a retrospective study, it is not possible to account for all the potential confounders, including cervical dilation at the time of induction and the practices of different providers. The sample size is small, and the data were collected at a single institution, which may make it difficult to generalize the results to other settings.

Cervical ripening methods differed between groups, with Foley balloon use being more common in patients with OUD (34% vs. 21%, *p* = 0.030), while misoprostol use did not differ significantly (3.8% vs. 7.6%, *p* = 0.2). Although Foley balloon use was more frequent in the OUD group, Foley balloon catheters are typically placed prior to amniotomy and therefore do not confound the amniotomy‐to‐delivery time interval. However, given that Foley balloon placement occurs within the total induction time window, we adjusted for Foley balloon use and age in the analysis of total induction time. After this adjustment, the difference in total induction time between groups was no longer significant (*p* = 0.105), while the amniotomy‐to‐delivery difference remained significant (*p* = 0.015). Additionally, while co‐exposure to xylazine and fentanyl has been documented since the early 2000s and was prevalent throughout our entire study period, xylazine testing was not routinely performed at our institution and its potential independent effect on myometrial contractility could not be assessed.

## 5 CONCLUSION

Patients with OUD did not require a higher total dose of oxytocin for induction compared to those without OUD. However, patients with OUD took significantly longer after amniotomy to deliver than those who were not using opioids. This finding suggests an association between opioid exposure and labor progression following amniotomy regardless of the amount of oxytocin used for induction. Further studies are necessary to clarify the physiologic reasons for the observed association and to help determine if the differences seen in the rate of labor progress would be clinically important enough to alter management decisions for patients undergoing labor induction.

## CONFLICT OF INTEREST STATEMENT

The authors declare no conflicts of interest.

## ETHICS STATEMENT

This study was reviewed and approved by the MetroHealth Medical Center Institutional Review Board. All data were collected through electronic medical records and de‐identified prior to analysis. The Institutional Review Board waived the requirement for individual patient consent due to the retrospective design of the study and use of de‐identified clinical data.

## Data Availability

The data that support the findings of this study are not publicly available due to institutional privacy policies but may be available from the corresponding author upon reasonable request and with permission of the MetroHealth Institutional Review Board.
